# The application of neutron imaging to examine ethene hydrogenation over a carbon-supported palladium catalyst

**DOI:** 10.1038/s41598-025-91179-6

**Published:** 2025-03-12

**Authors:** Hamish Cavaye, Christos E. Ballas, Winfried Kockelmann, Stewart F. Parker, Paul Collier, Andrew P. E. York, David Lennon

**Affiliations:** 1 ISIS Pulsed Neutron and Muon Source, STFC Rutherford Appleton Laboratory, Chilton, OX11 0QX UK; 2 School of Chemistry, University of Glasgow, Joseph Black Building, Glasgow, G128QQ UK; 3 Johnson Matthey Technology Centre, Blounts Court, Sonning Common, Reading, RG4 9NH UK

**Keywords:** Ethene hydrogenation, Pd/C catalyst, Neutron imaging, Reaction engineering, Imaging techniques, Catalysis

## Abstract

The combination of a heterogeneous catalyst operating within a defined reactor comes within the domain of reaction engineering, which takes cognisance of combined roles for both the catalyst and the reactor to define the overall operational system. One technique which is demonstrating much promise in investigating reaction engineering issues is neutron imaging. The technique is skewed towards monitoring hydrogen and hydrogenous species so, with hydrogen being ubiquitous in industrial organic chemistry and the penetrating power of the neutrons, neutron imaging can monitor hydrogen concentrations distributed throughout steel reactors whilst the reaction is taking place. In this way, neutron imaging can be used to assess the homogeneity of active catalyst beds and, additionally, determine how hydrogen is being partitioned throughout the catalyst bed as a function of time-on-stream. These are important parameters in the reaction engineering of catalytic systems involving transformations of hydrogen containing species. The article commences by reviewing the handful of existing neutron imaging studies in this field, then progresses to describe the application of the neutron imaging technique to investigate ethene hydrogenation over a 5 wt% Pd/C powder catalyst at 333 K and ambient pressure in a rectangular stainless-steel reactor. Modulations of the incident gas stream are seen to lead to spatially resolvable fronts moving across the bed and illustrate the diffusion of reagents from the reactor inlet across to the reactor exit. Thus, the investigation reveals spatially and temporally resolved elementary reactions that contribute to the hydrogenation process.

## Introduction

Levenspiel describes the concept of reaction engineering as “being concerned with the exploitation of chemical reactions on a commercial scale for purposes other than the production of power”^1^. When applied to heterogeneously catalysed reactions, reaction engineering requires a knowledge of the reactor and the catalyst that is facilitating the reaction. As such, the sub-discipline is at the interface of chemistry and chemical engineering. Within this domain, there is a driver for the application of techniques that can provide information on the composition and form of the catalyst bed all the way from the reactor inlet to the exit pipe whilst the reactor is ‘live’. In industrial scale packed bed reactors, thermocouples may be placed along the length of the reactor, so that the possibility of enthalpic changes throughout the length of reactor can be monitored^2^. Such arrangements can be useful in indirectly signalling the presence of ‘hot spots’, or defining catalyst deactivation profiles where, for example, the portion of the bed closest to the inlet deactivates first, but then the deactivating front progressively moves towards the reactor outlet as the reaction progresses^3^. This latter phenomenon, which has been compared to being analogous to that of a ‘burning cigar’^4^, can lead to a scenario where the catalyst at the front of the reactor is effectively ‘dead’, with product formation only occurring in the latter part of the reactor. Such events need to be carefully managed; for example, a knowledge of the deactivation kinetics enables a plant manager to predict when a new charge of catalyst will be required. How such important events can be monitored and studied is at the heart of reaction engineering.

Concerning the laboratory testing of reactor/catalyst formulation combinations involving microreactors and reactors able to contain multi-gram catalyst loadings, modern imaging and analytical techniques are providing much promise in guiding contemporary reaction engineering initiatives as enunciated above. For a conventional solid catalysed gas phase reaction (e.g. hydrogenation of an unsaturated hydrocarbon over a supported metal catalyst) imaging and analytical methods potentially provide the opportunity to assess, (1) uniformity of the packing of the catalyst bed, (2) gas/solid exchange dynamics, (3) the distribution of reagents/products throughout the length of the reactor, (4) the possibility of concentration gradients and (5) the presence of ‘hot spots’ or zones of inactivity. Access to such information could help assess catalyst/reactor configurations that deliver optimised and sustained product formation at commercially relevant rates.

X-ray imaging has considerable potential here^5^ but may put constraints on the choice of reactor materials; for example, due to strong absorption, stainless-steel reactors as used throughout the chemical manufacturing sector are problematical. Also, in terms of accessing information on the reactor contents, whereas X-rays are well suited to examining materials comprised of relatively heavy elements such as metal-based catalysts, they are unable to usefully probe the organic materials that typically comprise reagents and products. Conversely, the technique of magnetic resonance imaging (MRI) is well suited to analysing reagent/product combinations^6^ but it also encounters constraints with respect to suitable reactor materials (quartz reactors are fine, stainless-steel reactors are not). A further method for inspecting the functionality of a catalyst during active turnover with respect to reactor length is via the localised sampling of the gas phase by mass spectrometry utilising capillary sampling points evenly spaced between reactor inlet and outlet. For example, Sá and co-workers describe the use of a Spaci-MS system to examine the phenomenon of kinetic oscillations during a CO oxidation reaction over a Pt/Rh/Al_2_O_3_ catalyst supported on a cordierite monolith^7^. Collectively, these techniques are providing new information on reaction fronts and inhomogeneities within laboratory catalytic reactors.

Within the last few years, these methods have been supplemented using neutron imaging facilities that present considerable potential for exploring laboratory scale catalyst/reactor combinations where hydrogen (^1^H) is a significant component of the catalyst turnover process. The sensitivity to ^1^H, which is integral to the majority of catalytic processes, comes about from this isotope’s very high neutron attenuation cross section compared to other common elements^8^. In essence, neutron imaging involves the capability to determine how the concentration of hydrogen containing moieties may be perturbed over time and space. Neutron imaging is analogous to an absorption measurement, where increased concentrations of hydrogen lead to increased neutron attenuation that manifests itself as reduced transmission at a detector. Providing the incident neutron beam can be normalised so that the incident beam uniformly samples all the reactor volume, then the detected intensity is a function of hydrogen concentration within the detector pixel resolution. Due to the high penetrating power of neutrons, it is possible to visualise materials within stainless steel reactors. In this way, in principle, neutron imaging should be able to spatially determine hydrogen concentrations for a reacting system that is located within a stainless-steel reactor. Such an arrangement is helpful in advancing reactor engineering concepts.

There are only a small number of institutions that provide appropriate neutron imaging facilities. In 2016, Lehmann et al. reviewed the status of neutron imaging activities in a world-wide context and highlighted that there were only a dozen or so institutions with state-of-the-art neutron imaging facilities^9^. This limited global capacity of neutron sources means that there only a handful of neutron imaging catalytic reactor studies. Furthermore, it can be argued that neutron radiography does not necessitate any special neutron capabilities, however, enhanced scattering hydrogen cross sections at longer neutron wavelengths make cold neutron radiography beamlines particularly favourable for hydrogen detection. Against that background, it is informative to consider a few exemplar case studies to illustrate the capability and applicability of how these valuable facilities can be suitably deployed.

In 2019, Romanelli et al. used the IMAT neutron imaging instrument located at the ISIS Facility of the Rutherford Appleton Laboratory to undertake neutron radiography and tomography experiments on the *ortho*- to *para*-hydrogen conversion catalysed by a γ-Fe_2_O_3_ catalyst visualised within an aluminium cell^10^. The work showed how newly generated *para*-hydrogen poisons the catalyst, thus slowing the process and preventing the full conversion of large quantities of condensed molecular hydrogen. Subsequently, in 2023, Romanelli and co-workers used IMAT to image molecular-hydrogen adsorption and conversion in the HKUST-1 metal organic framework^11^. In 2020 Borgschulte and co-workers examined hydrogen in Cu/ZnO catalysts during methanol synthesis by neutron imaging using the cold neutron beamline ICON at the Swiss Neutron Spallation Source of the Paul Scherrer Institute (PSI) in Switzerland. The work was able to follow and quantify hydrogen containing species in Cu/ZnO catalysts during methanol synthesis^12^. Borgschulte and co-workers were also able to distinguish between irreversible hydrogen surface adsorption and reversible bulk absorption on a range of catalysts^13^. Recently, Borgschulte and co-workers have reviewed the benefits of combining neutron imaging with *operando* methods^14^. Then, in 2023 Cavaye and co-workers used IMAT to perform in situ real-time neutron imaging of gaseous H_2_ adsorption and D_2_ exchange experiments on carbon-supported Pd catalysts^15^. Although most measurements were undertaken in an aluminium cell with a Pd/C powder catalyst, preliminary data was presented for H_2_/D_2_ exchange over a pelleted Pd/C catalyst contained within a flat stainless-steel cell. The favourable results observed open the way for the investigation of reactions at elevated temperatures and pressures.

This article builds directly on the previously reported Pd/C-H_2_/D_2_ adsorption/desorption/exchange studies^15^ to use neutron imaging to investigate a real-time chemical reaction, namely ethene hydrogenation over a powdered 5 wt% Pd/C catalyst at 333 K and ambient pressure contained within a stainless-steel reactor. Ethene hydrogenation can be considered as a ‘benchmark’ reaction in heterogeneous catalysis^16–19^. The successful introduction of a steel reactor and a lower, more industrially relevant Pd-loading, without a loss of sensitivity, directly demonstrates the capability of the technique to be utilised at high temperatures and with the possibility of future work at higher pressures. (The cell used in this work is designed for use up to 400 °C and 5 bar). The article will examine how hydrogen partitions throughout the catalyst bed as the hydrogen and ethene flow rates are varied. Changes in neutron attenuation as a function of space and time then provides information on the relative rates of hydrogen diffusion throughout the bed on experiencing such perturbations in gas delivery.

## Experimental

### Catalyst and reaction conditions

8.2 g of a 5 wt% Pd/C powder catalyst (Sigma-Aldrich, Product # 205680) were loaded into a flat stainless-steel cell (dimensions of sample-relevant part approx. 48 width × 65 height × 17 depth mm, outer dimensions). Electrical Kapton film heaters (adhesive backed, 10 × 155 mm, 28 V, 25 W) and temperature sensors were attached to the top and bottom flanges of the cell for temperature control. The base of the cell was lined with quartz wool (Elemental Microanalysis) then the catalyst loaded into the cell. As part of the catalyst charging process, the reactor was regularly tapped and the catalyst pressed with a spatula, so that the bed was evenly compressed. A layer of quartz wool was then packed in at the top of the bed and the reactor sealed. The cell contained Swagelok connections which allowed gases to enter through the bottom of the cell, flow through the sample, and exit at the top. Traces of water were removed from the catalyst prior to use on the IMAT beamline via heating at 200 °C for 3 h under vacuum. The vacuum was supplied via a turbomolecular pumping station that was connected through the top of the cell.

During beamtime operation, the cell was connected to a gas handling panel through approximately 6 m of 1/8″ diameter stainless-steel line. The exit line of the reactor was connected to an extraction system. The gas panel was located outside the IMAT experimental beam area and equipped with the following gases: He (Air Liquide, 99.999%), H_2_ (Compressed Gas Solutions Ltd, 99.9995%), D_4_-Propyne (Cambridge Isotope Laboratories, C_3_D_4_, 98%) and Ethene (BOC, 99.9%). Gas flow rates were individually controlled through a series of HFC-302 Teledyne mass flow controllers connected to Chell CCD100 Controller boxes. The timescale for gas switching events was 1–2 s at the gas manifold but the time taken for such an event to be registered at the exit line for an empty cell was approximately 2 min.

### Neutron radiography

Neutron radiography was performed at the IMAT beamline^20^, using a polychromatic neutron beam of wavelength range 0.7–6.7 Å. The neutron flux at the sample position was of the order 10^7^ n cm^−2^ s^−1^. The sample cell was mounted 45 mm in front of a ZnS/LiF:Cu scintillator screen (100 μm thickness) of a neutron camera box. The scintillator screen was at a distance L = 10.4 m from a beam aperture of diameter D. The scintillator was coupled via a 45-degree mirror and a focussing lens of 85 mm focal length to a CMOS camera (ANDOR Zyla sCMOS 4.2, Oxford Instruments, UK) with 2048 × 2048 pixels, providing a field-of-view of 112 × 112 mm^2^ and a pixel size of 55 × 55 μm^2^. With a beam aperture of D = 100 mm and an L/D ratio of about 100 the geometric blur and resolution limit was about 450 μm.

### Data processing procedures

Image frames were recorded with a 5 s exposure time on a loop. With approximately 1–2 s of overhead time for the data to save between frames, the frame acquisition rate was approximately 1 frame every 6–7 s. Within the frame work-up procedure, the following calibration workflow was adopted: the detector output images were loaded in to the Mantid imaging software package^21,22^; open beam (flat field) and dark calibration runs were applied (including removal of bright and dark outliers); a direct beam region of interest (ROI) scaling was applied to correct for variations in beam intensity that exist within a run; data were converted to 16 bit and saved to disk. Image manipulation for animations was performed using the ImageJ/Fiji software package^23^ and saved as a stacked *.tif file. To follow how a particular perturbation (e.g. change in gas composition at time t) affected the reactor image, a median average frame was created from the first 49 frames of a run, prior to any perturbation being imparted, and saved as a reference image. In this way, changes in the image as a function of time-on-stream were referenced to this averaged value (normalised intensity, I_0_ = 1.000 au). This process of normalisation was necessary due to large absolute grey value differences in each region of interest (ROI) being investigated, e.g. the grey values for the air regions outside of the reactor were substantially larger than for those inside the reactor. Normalisation in this manner allowed the relative changes in intensity during any perturbation to be compared across the catalyst bed.

Once these pre-processing steps had been completed, a custom-written Python script was used to further analyse the runs; specifically, to examine the changes in neutron intensity (grey value) of various ROIs as the run progressed. The ROIs used were 100 × 100 pixels (5.5 × 5.5 mm) in size. This was chosen because empirically it offered a balance between being large enough that the grey value plots had a reasonable noise level, while also being small enough to afford good spatial differences around the sample. A systematic study into the effect of the ROI size was out of the scope of this work. A Savitzky-Golay smoothing algorithm was then applied to the resulting grey value lines to aid in the clarity of the plots. Finally, each interrogated ROI was colour-coded to match the line in the grey value plot. These ROIs were chosen to investigate a variety of locations in the catalyst bed, with areas external to the reactor (as signified by black and blue squares in the figures) acting as internal references.

Three parameters were used to define the characteristics of the trends observed: (1) the % change in grey value intensity (δGV, %) observed on initiation of a perturbation event relative to the normalised pre-perturbation value (i.e. related to change in neutron attenuation); (2) the half-life (t_½_, min) of the intensity change following the perturbation event (i.e. an indicator of the time taken to achieve the post-perturbation grey value); and (iii) the lag time (δt_max_, min) that represents the maximum time period between observing changes in grey value intensity for different regions of the reactor (i.e*.* an indicator of the time taken for a reaction front to be experienced by the whole reactor).

δGV are reported here with a precision of 0.05–0.1%, which was calculated by interrogating the standard deviation of GV for each ROI in each measurement during a steady-state portion of the experiment. These values are then used to calculate the uncertainty values given in Table 1.

## Results

### Catalyst activation

**Fig. 1:**
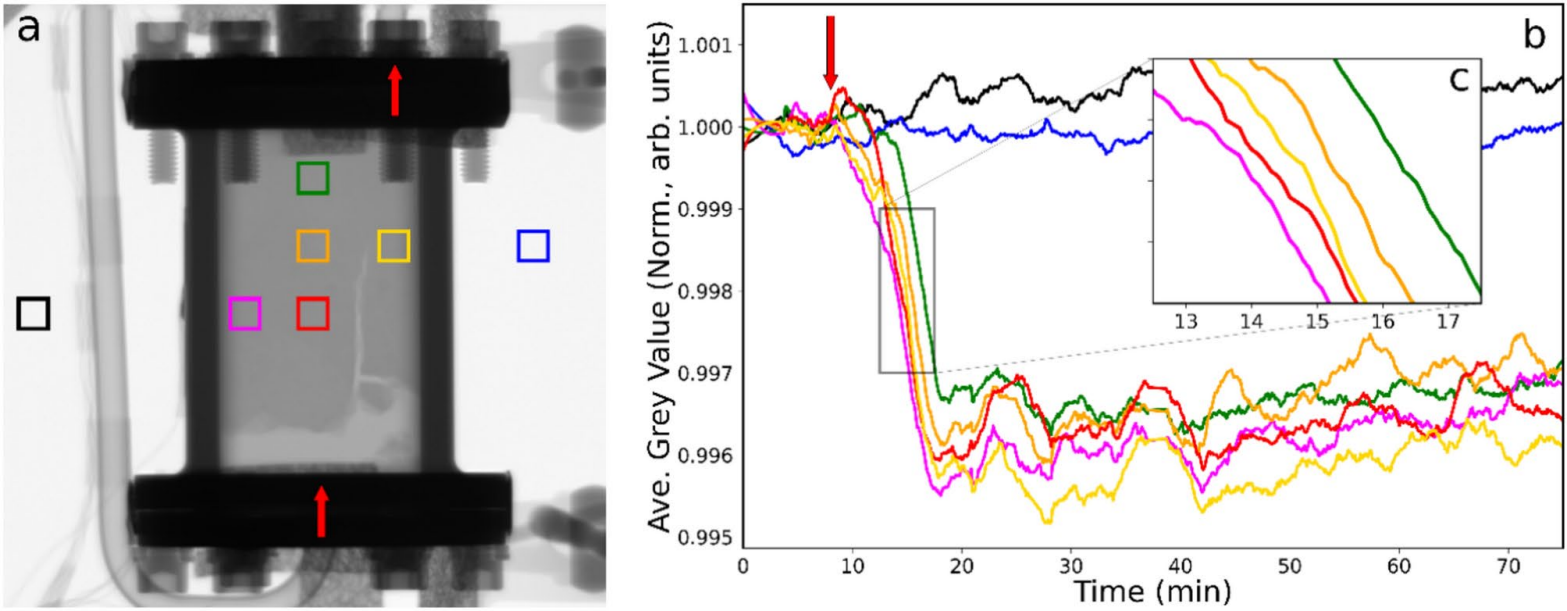
(**a**) Neutron radiograph of the catalyst contained within the reactor at 298 K under conditions of continuous helium flow (100 ml min^−1^) when hydrogen (20 ml min^−1^) is switched into the feedstream. The squares define regions of interest (ROI) inside and outside of the reactor where neutron intensity is monitored as a function of time. The bottom and top red arrows denote the gas flow inlet and outlet positions, respectively. (**b**) Neutron intensity (defined as grey value) versus time plot for the colour coded ROIs. The arrow indicates the time at which hydrogen was introduced to the incident gas feedstream. (**c**) The inset zooms in on the time period corresponding to the maximum change in greyscale.

Figure 1a presents a neutron radiograph of the reactor filled with catalyst at 298 K as helium is passed over the catalyst at 100 ml min^−1^. Gas entry is via the pipe connected to the bottom flange, with the exit line located at the top of the reactor as viewed. The grey region that fills the top 80% of the reactor is the catalyst powder, whilst the lighter region at the base is quartz wool that was used as a base when charging the catalyst. Quartz wool also sits on top of the catalyst bed but is not visible in the image, as it is behind the highly attenuating cell flange. Towards the bottom right-hand side of the reactor a white ‘stream’ is visible. This is due to an ‘opening up’ of the bed at the base, which presumably occurred during initiation of the helium gas flow. The seven squares define the regions of interest (ROI) selected for inspection throughout the study. The black and blue squares are reference points located outside of the reactor, whereas the five squares within the reactor define different regions within the catalyst bed. ROIs were chosen to correspond to parts of the catalyst bed that did not undergo significant shifts in packing density during each respective run. Changes in the packing density of the catalyst can lead to dramatic changes in the neutron attenuation, thus overwhelming the signal from changes in gas adsorption.

Figure 1b shows curves corresponding to the average grey values in each ROI for each frame in the collected datasets over time, with the arrow indicating the time that hydrogen is switched on (20 ml min^−1^) and entrained within the He carrier stream. Through the period studied (75 min), the grey values for the two reference ROIs remain at a constant level. However, in contrast, on hydrogen addition, all five of the ROI located within the reactor display a rapid decrease in intensity, due to hydrogen adsorption increasing neutron attenuation, while thereafter the grey value changes (δGV) level out at approximately − 0.4% of the reference value. The decay of grey value evident in Fig. 1b corresponds to a t_½_ value of 6–8 min. Figure 1c shows a close-up of the decay profile that reveals a maximum time lag (δt_max_) for when different parts of the reactor experience the incoming hydrogen gas. For instance, the magenta ROI located to the middle left of the reactor ‘sees’ the hydrogen 3–4 min before that sensed in the green ROI that is located at the top of the reactor. This time difference represents the time taken for the hydrogen to diffuse within different regions of the catalyst bed.

**Fig. 2:**
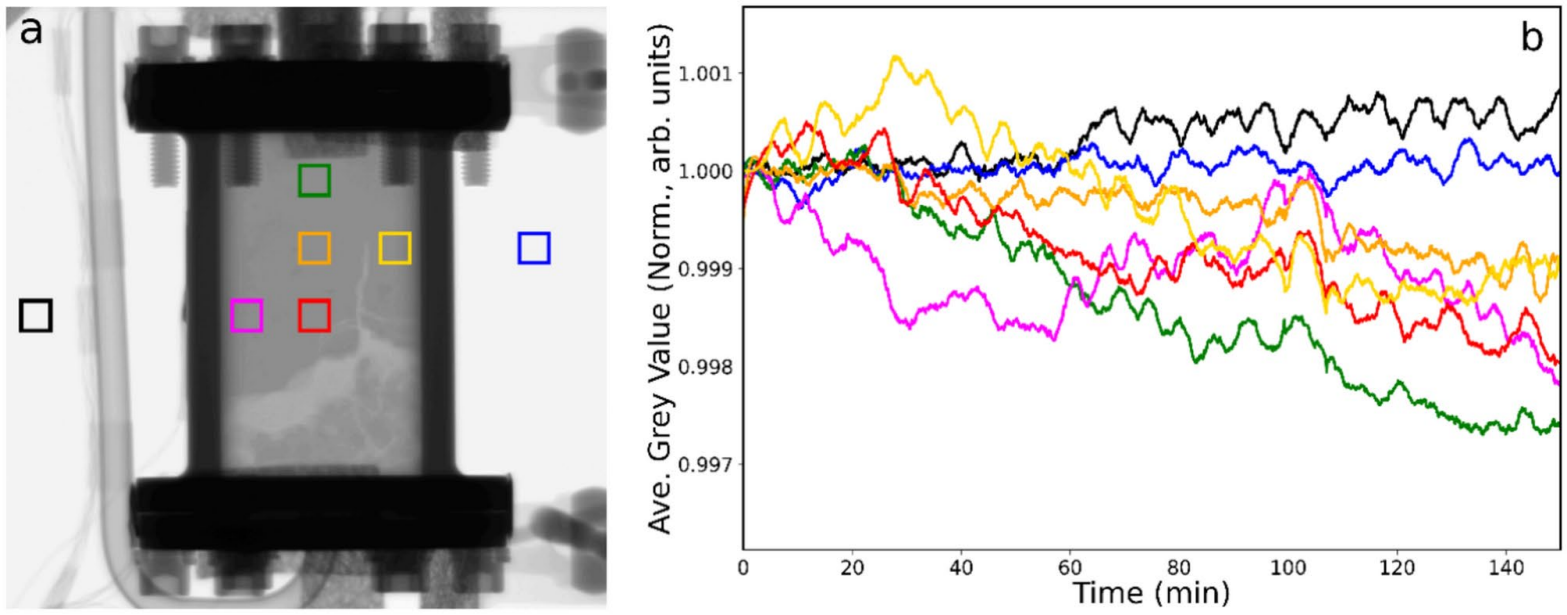
(**a**) Neutron radiograph of the catalyst contained within the reactor at 298 K under conditions of continuous helium (100 ml min^−1^) and hydrogen (20 ml min^−1^) flow when the temperature is increased to 333 K over a period of 2 h. (**b**) Neutron intensity (defined as grey value) versus time plot for the colour coded ROI. Heating commenced at t = 0.

In our original measurements, the Pd/C catalyst was activated adopting an ex-situ arrangement^15^. As part of method development, for this study the reactor was fitted with heaters and temperature sensors to facilitate in-situ activation. Whilst maintaining the hydrogen flow, Fig. 2a shows a neutron radiograph of the reactor as the temperature is progressively increased to 333 K over a period of 2 h. Figure 2b shows the corresponding greyscale intensities over a 2.5 h period. Comparison between Figs. 2a and 1a shows that the warming process has caused further disruption to the packed bed, as indicated by some movement of catalyst in with the quartz wool base and some widening of the channel located to the middle-right of the reactor. Figure 2b shows a degree of discontinuous behaviour for different parts of the bed that is connected with a slight degree of movement of the reactor during the warming process. Frames were aligned before data analysis to account for reactor movements, however, such corrections can only account for translations in the plane of the image. Any level of cell rotation or twisting out of the plane of the image cannot be corrected for and may lead to the observed discontinuities. The small change in greyscale value (δGV) observed suggest a small degree of additional hydrogen uptake on heating the catalyst.

### Hydrogen/ethene co-feed

Supported metal catalysts applied to hydrogenation reactions can exhibit a catalyst conditioning stage in advance of achieving steady-state operation^24^. In order to ensure that our neutron imaging measurements better reflect a stabilised catalyst, a flow of hydrogen and propyne (3:1 ratio) was passed over the catalyst maintained at 333 K for 24 h. Thereafter the propyne and hydrogen was switched off and He passed over the catalyst for 4 h. This treatment left the catalyst in an active and stabilised state, ensuring that subsequent reactions represented the hydrogenation activity over a ‘mature’ catalyst.

**Fig. 3:**
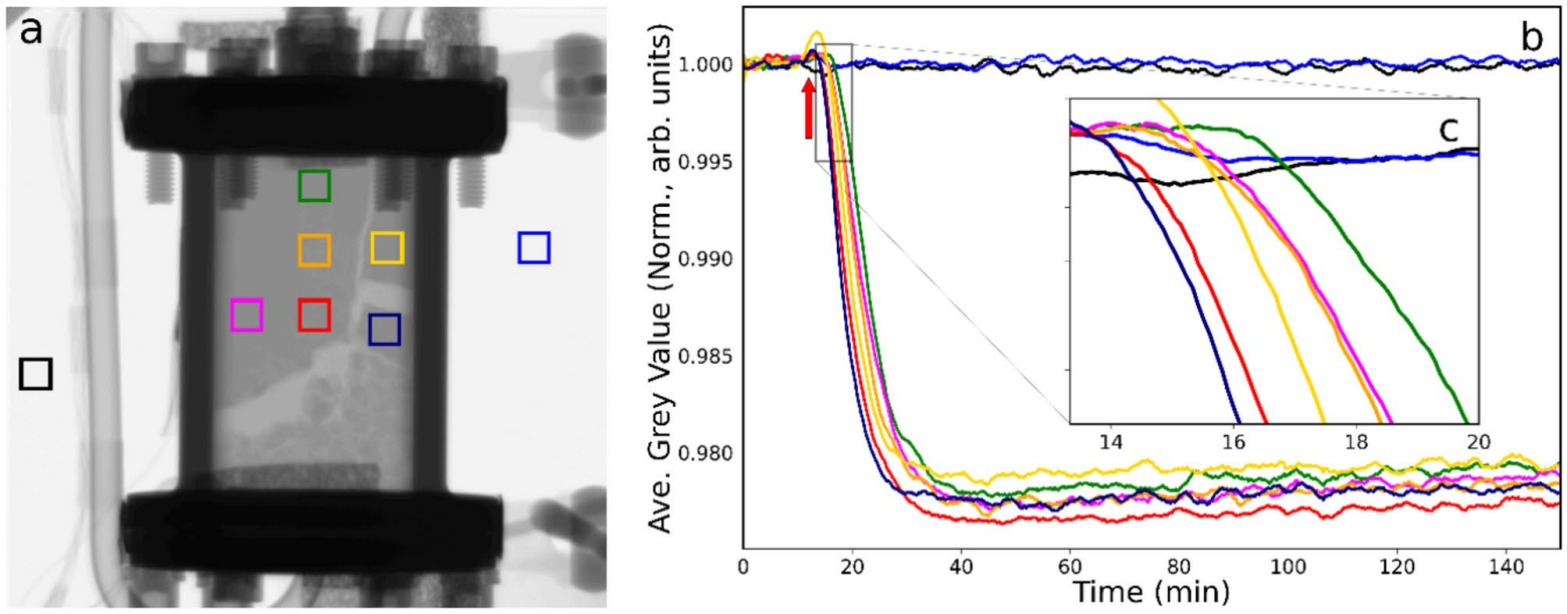
(**a**) Neutron radiograph of the catalyst contained within the reactor at 333 K under conditions of continuous helium (100 ml min^−1^) and hydrogen (5 ml min^−1^) flow when ethene (5 ml min^−1^) is switched into the feedstream. (**b**) Neutron intensity (defined as grey value) versus time plot for the colour coded ROIs. The arrow indicates the time at which the ethene was introduced into the incident gas feedstream. (**c**) The inset provides a zoomed view of the time period of maximum grey scale changes.

Figure 3 presents the neutron imaging dataset for the catalyst as ethene (5 ml min^−1^) was entrained within the He (100 ml min^−1^) and hydrogen (20 ml min^−1^) co-feed, with the reactor maintained at 333 K. Figure 3a shows further levels of separation of the packed column within the bottom right side of the reactor, with a channel now evident in the top right-hand quadrant. The left middle and top of the bed remained compressed and appear effectively homogeneous as indicated by an even greyness in this region. It is worth noting that prior to the introduction of ethene, a shift in the packing of the catalyst bed occurred and thus the ROIs chosen in Fig. 3 (and for the remainder of this work) are slightly different to those in Figs. 1 and 2 and include an additional eighth ROI in navy blue.

The arrow in the grey value temporal profile (Fig. 3b) indicates the time at which the ethene was switched into the incident gas feedstream, which leads to a sharp and relatively intense decrease in the grey value (δGV = − 2.4% of the reference value) for the reactor ROIs. The decay observed corresponds to a t_½_ value of 13–17 min, whilst Fig. 3c reveals a time lag between the blue and green ROIs of δt = 3–4 min. Thus, the decrease in grey value is significantly greater compared to when hydrogen was introduced to the catalyst (Fig. 1b), due to the greater number of hydrogen atoms in ethene, however, the t_½_ value shows that it has taken longer for the ethene to diffuse through the bed compared to the hydrogen. The final grey values beyond the decay remain constant at broadly comparable grey values, indicating hydrogenation to be occurring to a similar degree throughout the whole reactor volume.

**Fig. 4:**
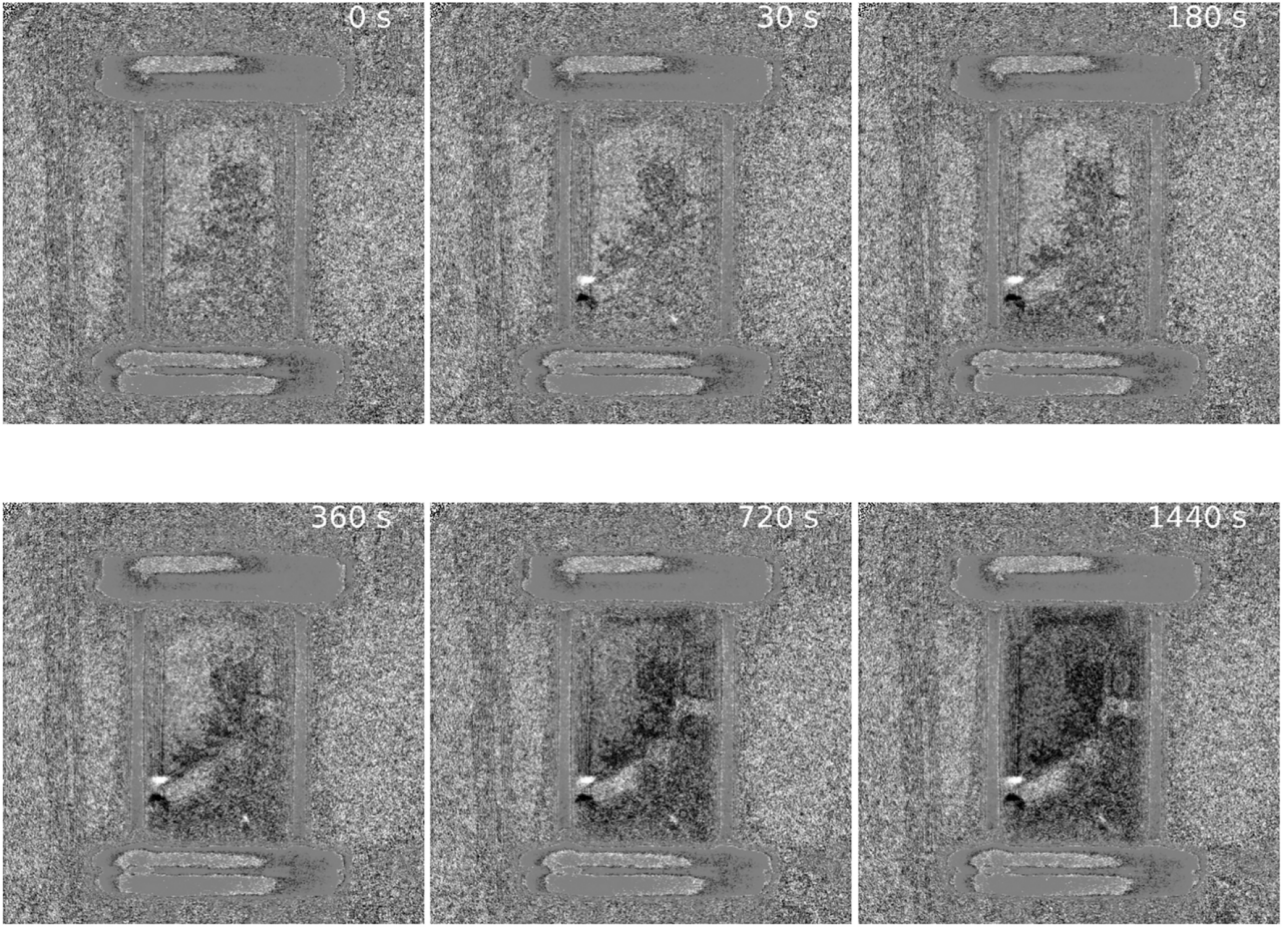
A selection of frames spanning approximately 24 min over the period when the ethene was switched into the He/H_2_ gas feed (Fig. 3b). A small shift in the catalyst bed in the lower-left region of the cell, responsible for the large white/black marks, can be ignored.

To investigate differences in the onset of adsorption across the catalyst bed, a difference image movie was generated by subtracting the initial state reference frame from the entire image stack. Figure 4 presents a selection of frames spanning approximately 24 min and reveals the progression of a diffusion front of grey value reduction over this period. In a similar manner to that reported previously for hydrogen adsorption over a 20 wt% Pd/C catalyst^15^, a decrease in grey value spreads out from the lower part of the cell, and from the edges of the cracks in the catalyst bed. A video of this introduction of the ethene to the He/H_2_ co-feed to the conditioned catalyst is presented in the Supporting Information section (SVideo-1).

### Varying hydrogen and ethene flow rates

Two groups of measurements were undertaken to assess the catalyst’s response to, firstly, a relative decrease in the flow of hydrogen gas then, secondly, an increase in ethene flow rate for a fixed hydrogen flow rate.

**Fig. 5:**
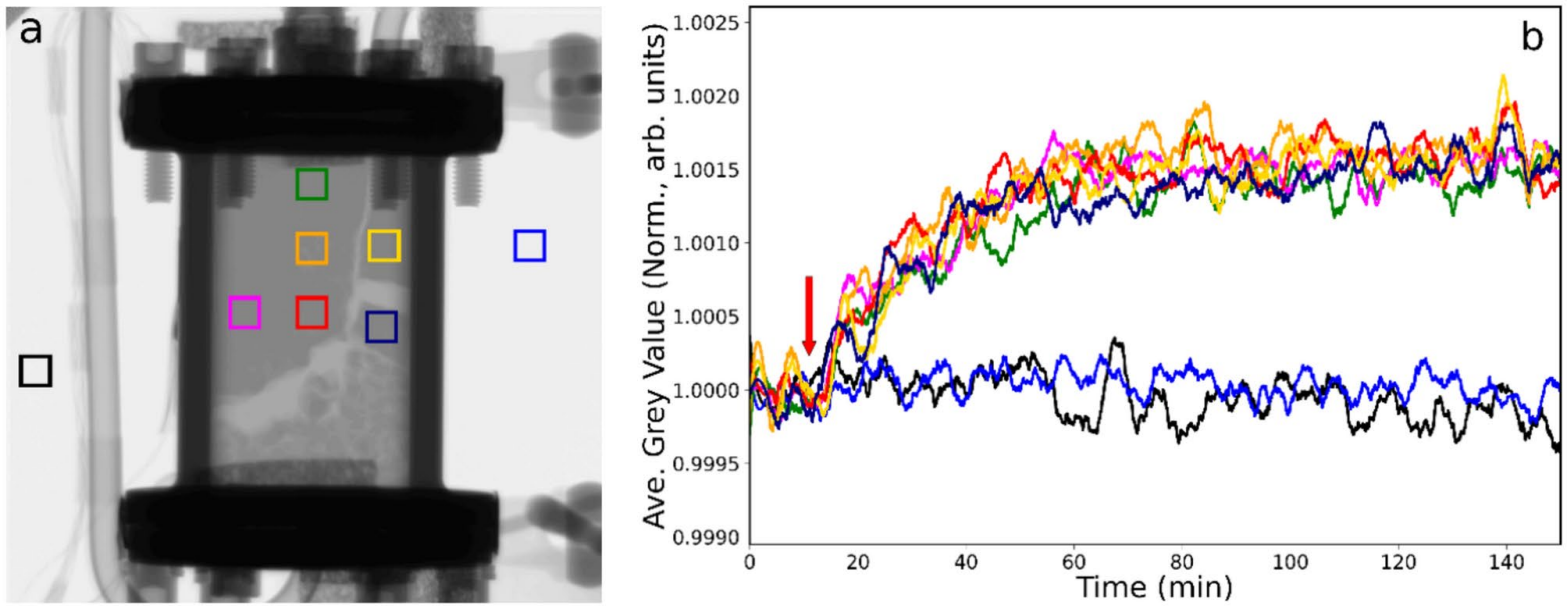
(**a**) Neutron radiograph of the catalyst contained within the reactor at 333 K under conditions of continuous helium (100 ml min^−1^), hydrogen (20 ml min^−1^) and ethene (5 ml min^−1^) flow when the hydrogen flow rate was reduced to 5 ml min^−1^. (**b**) Neutron intensity (defined as grey value) versus time plot for the colour coded ROIs. The arrow indicates the time at which hydrogen in the incident gas feedstream was reduced.

In the first instance, the catalyst was run in a hydrogen-rich regime (He 100 ml min^−1^, H_2_ 20 ml min^−1^, C_2_H_4_ 5 ml min^−1^) and the normalised grey value used as a reference (I_0_ = 1.000). Figure 5 shows a neutron radiograph of the reactor (Fig. 5a) and the temporal profiles of the associated regions of the reactor at the point when the hydrogen flow rate was reduced to 5 ml min^−1^ (Fig. 5b), i.e. the catalyst was abruptly tipped into a hydrogen-lean regime. Figure 5b shows a relatively slow increase in grey value, equalising at a δGV value of approximately + 0.15% relative to I_0_., with the positive value signifying a loss of hydrogen from the catalyst matrix. Whereas no discernible time lag between regions of the reactor is evident for this gradual loss of hydrogen, a t_½_ value of 50–60 min shows the hydrogen depletion process under hydrogenation conditions to be much slower than the hydrogen adsorption process evidenced in Fig. 1b.

**Fig. 6:**
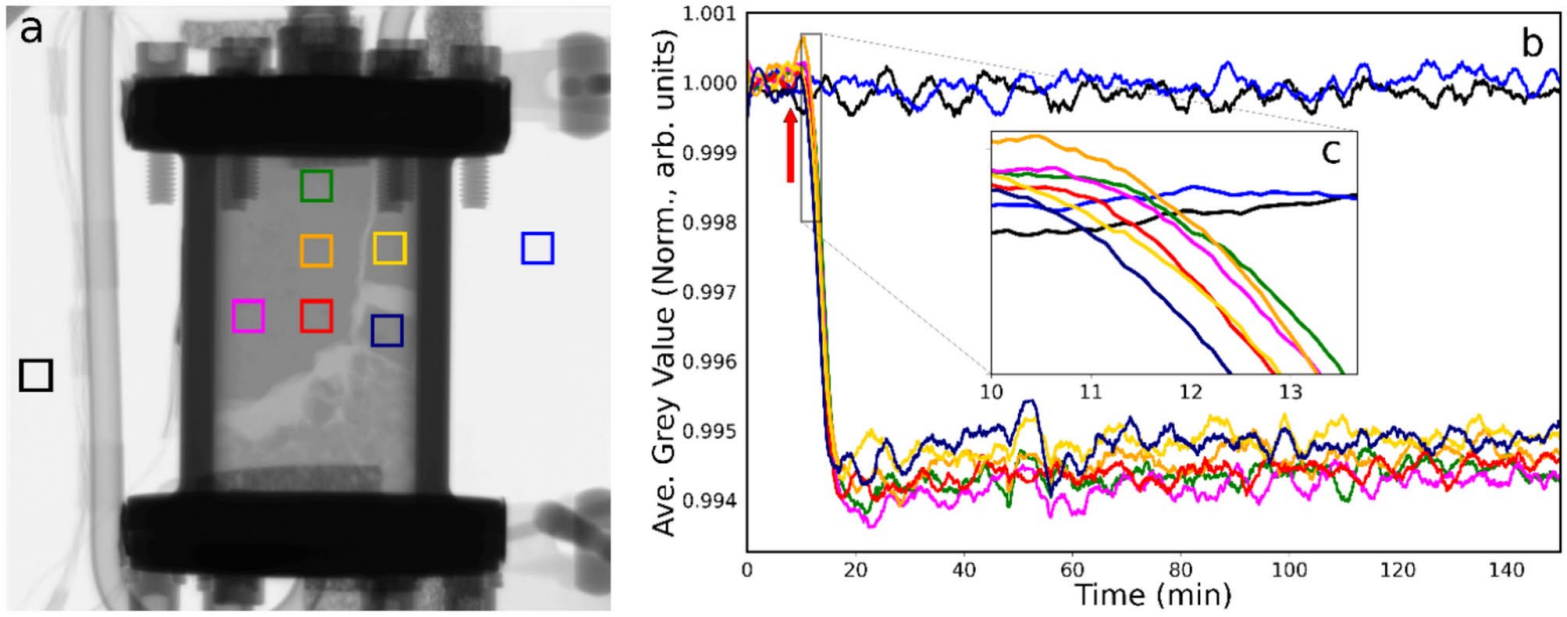
(**a**) Neutron radiograph of the catalyst contained within the reactor at 333 K under conditions of continuous helium (100 ml min^−1^), hydrogen (5 ml min^−1^) and ethene (15 ml min^−1^) flow when the ethene flow rate was increased to 25 ml min^−1^. (**b**) Neutron intensity (defined as grey value) versus time plot for the colour coded ROIs. The arrow indicates the time at which ethene in the incident gas feedstream was increased. (**c**) The inset zooms in on the time period of maximum greyscale changes.

Secondly, the catalyst was run in a hydrogen-lean regime (He 100 ml min^−1^, H_2_ 5 ml min^−1^, C_2_H_4_ 15 ml min^−1^) and, as above, the normalised grey value used as a reference (I_0_ = 1.000). Figure 6 shows a neutron radiograph of the reactor (Fig. 6a) and the temporal profile of the associated regions of the reactor at the point when the ethene flow rate is increased to 25 ml min^−1^ (Fig. 6b) i.e. the catalyst was abruptly tipped into an ethene-rich regime with the olefin in a 5:1 volumetric excess. Like that observed in Fig. 3b, Fig. 6b is characterised by a decrease in grey value (δGV = − 0.6% relative to I_0_), with the negative value indicating that more ethene was being adsorbed at the surface. However, the magnitude of the adsorption is less than observed in Fig. 3b (δGV = − 2.4%) suggesting that the ethene coverage was approaching saturation. This process is relatively rapid, with the decay in Fig. 6b exhibiting a t_½_ value of *ca*. 5 min (*c.f*. 13–17 min in Fig. 3b). The rapidity of the ethene adsorption process is also reflected in the maximum time lag (δt_max_) between different zones being approximately 1 min.

On comparison of the neutron radiographs presented in Figs. 3a and 5a, it is seen that the structure of the packed catalyst bed is similar in both images. Indeed, following the introduction of the hydrogen/ethene co-feed at elevated temperature described in Sect. 3.2 (Fig. 3a), thereafter the catalyst bed remained essentially stable. Thus, it appears that after the initial packing of the powdered catalyst, the introduction of flowing gases and warming events can compromise the homogeneity of the bed by movement of sections of the bed and a degree of channelling but, after some compression, the structure of the bed is stabilised.

### Stopping the hydrogen flow whilst maintaining ethene flow

**Fig. 7:**
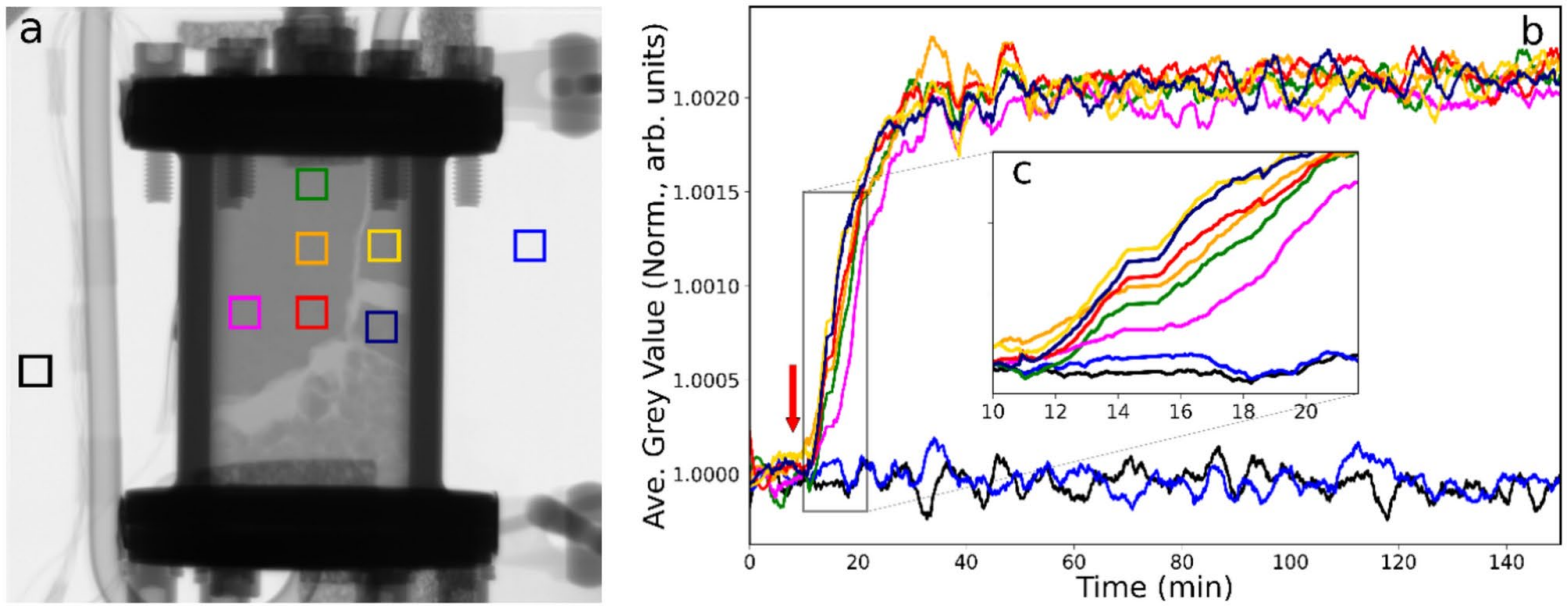
(**a**) Neutron radiograph of the catalyst contained within the reactor at 333 K under conditions of continuous helium (100 ml min^−1^), hydrogen (5 ml min^−1^) and ethene (25 ml min^−1^) flow when the hydrogen flow rate was stopped. (**b**) Neutron intensity (defined as grey value) versus time plot for the colour coded ROIs. The arrow indicates the time at which hydrogen in the incident gas feedstream was stopped. (**c**) The inset zooms in on the time period corresponding to the maximum greyscale changes.

Further stressing the catalyst, Fig. 7 presents the neutron radiograph of the reactor (Fig. 7a) and the associated neutron transmission versus time curves (Fig. 7b) during a period when the hydrogen supply was stopped. To the left-hand side of the arrow, the neutron intensity has been normalised for the preceding hydrogen-lean flow conditions (He 100 ml min^−1^, H_2_ 5 ml min^−1^, C_2_H_4_ 25 ml min^−1^), I_0_. At the run time indicated by the arrow (t = *ca*. 6.7 min), the hydrogen flow was stopped. Figure 7b shows a progressive increase in grey scale, associated with a reduction of hydrogen within the catalyst matrix, which then plateaus in intensity.

The magnitude of the intensity change (δGV) at 333 K is + 0.2%, which is half of that observed for the hydrogen up-take process at 298 K (− 0.4%, Fig. 1b). As Fig. 2b showed an, albeit sporadic, increase in retained hydrogen on warming to 333 K (~ − 0.2%, Fig. 2b), the intensity changes are thought to indicate that the loss of hydrogen from the catalyst matrix once hydrogen flow was stopped was not entirely reversible under the conditions studied. We note that these changes discussed are relative changes, as mentioned in the description of the grey value normalisation process above. However, this method of comparison between perturbations remains semi-quantitative and the conclusions are justified.

## Discussion

Table 1 presents the intensity change values (δGV), half-lives (t_½_) and lag times (δt_max_) for Figs. 1, 2, 3, 5, 6 and 7 and indicates that several processes are contributing to the ethene hydrogenation process, with different processes exhibiting different spatial and/or temporal characteristics. For example, the lag time parameter (δt_max_) is a useful indicator for how diffusion is affecting hydrogen partitioning throughout the bed. Although a time-lag of approximately 4 min does exist between regions of the bed close to and far away from gas exchange channels, overall, the physical diffusion throughout the bed is even and efficient, with homogeneous concentrations of hydrogenous species achieved within a 4 ± 1 min time window.

Concerning processes which are more chemical in origin, the growth curve in Fig. 7b exhibits a t_½_ value of 20 min, which indicates the hydrogen depletion to be faster when the hydrogen supply is abruptly stopped compared to that observed when the hydrogen flow rate is simply diminished (t_½_ = 50–60 min, Fig. 5b). Moreover, a δGV value of + 0.2% for the extended plateau region of Fig. 7 is well within the range of values seen for the perturbations explored in this study (Table 1) and indicates that, despite there being no dihydrogen flow, there is nonetheless a significant population of hydrogenous species retained by the catalyst. Figure 7 shows this residual hydrogen to be evenly distributed throughout the catalyst bed (δGV = + 0.2 ± 0.02% for all six ROIs shown in Fig. 7a). In the extended absence of a hydrogen supply, no hydrogenation activity is tenable, so what is the nature of the species responsible for the neutron absorption evident in Fig. 7? As noted by Borgschulte et al.^12^, despite many positive attributes, neutron imaging is hindered by an inability to spectroscopically probe the materials under examination. In our initial study on H_2_/D_2_ adsorption and desorption over Pd/C, inelastic neutron scattering measurements of the reactor post-reaction demonstrated the presence of β-PdH^15^. Whilst hydride species could indeed be contributing to the profile displayed in Fig. 7, it is thought more likely that it represents a hydrocarbonaceous overlayer^25^ that was facilitating the facile hydrogenation process. More work is required to explore this matter further.

**Table 1:** Neutron greyscale change (δGV) relative to the normalised intensity (I_0_), half-life of decay progressing towards to a new equilibrium value (t_½_) and maximum lag time (δt_max_) between different parts of the reactor on initiation of a defined perturbation.

	Conditions (perturbation following I_0_)	Temperature (K)	Intensity change relative to I_0_ (δGV, %)	Half life (t_½_, min)	Lag time (δt, min)
1	Hydrogen adsorption [He 100 ml min^−1^] → [He 100 ml min^−1^ + H_2_ 20 ml min^−1^]	298	− 0.4 ± 0.13	6–8	3–4
2	Heating cell [He 100 ml min^−1^] + [H_2_ 20 ml min^−1^]	298–> 333	(− 0.1) to (− 0.3)^a^	–	–
3	Introducing ethene [He 100 ml min^−1^ + H_2_ 5 ml min^−1^] → [He 100 ml min^−1^ + H_2_ 5 ml min^−1^ + C_2_H_4_ 5 ml min^−1^]	333	− 2.4 ± 0.13	13–17	3–4
5	Reducing hydrogen flow [He 100 ml min^−1^ + H_2_ 20 ml min^−1^] + C_2_H_4_ 5 ml min^−1^] → [He 100 ml min^−1^ + H_2_ 5 ml min^−1^ + C_2_H_4_ 5 ml min^−1^]	333	+ 0.15 ± 0.08	50–60	–
6	Increasing ethene flow [He 100 ml min^−1^ + H_2_ 5 ml min^−1^ + C_2_H_4_ 15 ml min^−1^] → [He 100 ml min^−1^ + H_2_ 5 ml min^−1^ + C_2_H_4_ 25 ml min^−1^]	333	− 0.6 ± 0.11	5	1
7	Stopping hydrogen flow [He 100 ml min^−1^ + H_2_ 5 ml min^−1^] + C_2_H_4_ 25 ml min^−1^ → [He 100 ml min^−1^ + H_2_ 0 ml min^−1^ + C_2_H_4_ 25 ml min^−1^]	333	+ 0.2 ± 0.05	20	5

^a^No uncertainty was calculated for this run due to the movement of the cell during heating. These are approximate values only.

## Conclusions

Neutron imaging has been used to examine, firstly, the adsorption of hydrogen over a conditioned 5 wt% Pd/C catalyst housed in a stainless-steel reactor then, secondly, ethene hydrogenation at 333 K and ambient pressure has been explored. Repeated scanning as the reaction system experienced a defined perturbation brought about by rapidly changing gas composition has been informative in delineating various physical and chemical processes. Despite neutron radiographs revealing fractures and distortions within the catalyst bed, physical diffusion of gaseous reagents promptly (4 ± 1 min) led to even concentrations of hydrogenous species throughout the bed. Conversely, switching from a hydrogen-rich to a hydrogen-lean regime was seen to lead to a slow re-equilibration of the reaction system (t_½_ = 50–60 min). Thus, this neutron imaging investigation is revealing temporal distinctions of elementary reactions that contribute to the hydrogenation process. Moreover, it is showing how they are spatially partitioned throughout the whole reactor volume. During a process of maintained ethene supply and starvation of a hydrogen feed, the presence of retained hydrogenous moieties is attributed to a hydrocarbonaceous overlayer that, until the hydrogen was stopped, was facilitating hydrogenation activity.

This work demonstrates that it is possible to follow an industrially relevant reaction in real time at realistic temperatures. As such, it is a significant advance on our initial study that followed H_2_/D_2_ adsorption/desorption using a cell and catalyst that were as optimal as possible for the experiment. The absence of spectroscopic information is a clear drawback of the technique and we are actively looking at ways to mitigate this problem.

## Supplementary Information

Supplementary Information 1.

Supplementary Information 2.

Supplementary Information 2.

Supplementary Information 2.

## Data Availability

In addition to the supporting information, all raw image datasets generated and/or analysed during the current study are available in the ISIS data DOI for ISIS experiment RB2310426 repository, accessible via Ref.^26^.

## References

[1] Levenspiel, O. *Chemical Reaction Engineering: An Introduction to the Design of Chemical Reactors* (Wiley, 1962).

[2] https://blog.wika.us/knowhow/thermocouple-sensors-catalyst-die-off-predictions/ (accessed 22nd September 2024).

[3] P. Bollini & A. Bhan, Deactivation mechanisms in methanol-to-hydrocarbons chemistry, *Catalysis*, **30**, 146–156 (2018).

[4] Haw, J. F. & Marcus, D. M. Well-defined (supra) molecular structures in zeolite methanol-to-olefin catalysis. *Top. Catal.* **34**, 41–48 (2005).

[5] Errigo, M., Lettieri, P. & Materazzi, M. X-ray imaging techniques for gas-solid fluidized beds—A technical review. *Particuology.*10.1016/j.partic.2023.11.013 (2023).

[6] Zheng, Q. et al. Operando magnetic resonance imaging of product distributions within the pores of catalyst pellets during Fischer-Tropsch synthesis. *Nat. Catal.* **6**, 185–195 (2023).

[7] Sa, J. et al. SpaciMS: Spatial and temporal operando resolution of reactions within catalytic monoliths. *Analyst* **135**, 2260–2272 (2010). 20697617 10.1039/c0an00303d

[8] Yu, X. et al. Neutron scattering studies of heterogeneous catalysis. *Chem. Rev.* **123**, 8638–8700 (2023). 37315192 10.1021/acs.chemrev.3c00101 PMC10347434

[9] Lehmann, E., Trtik, P. & Ridikas, D. Status and perspectives of neutron imaging facilities. *Phys. Procedia* **88**, 140–147 (2017).

[10] Romanelli, G. et al. Visualization of the catalyzed nuclear-spin conversion of molecular hydrogen using energy-selective neutron imaging. *J. Phys. Chem. C* **123**, 11745–11751 (2019).

[11] Simoni, M., Minniti, T., Senesi, R. & Romanelli, G. Molecular specificity in neutron imaging: The case of hydrogen adsorption in metal organic frameworks. *Phys. Chem. Chem. Phys.* **25**, 30821 (2023). 37961753 10.1039/d3cp04176j

[12] Terreni, J. et al. Hydrogen in methanol catalysts by neutron imaging. *Phys. Chem. Chem. Phys.* **22**, 22979–22988 (2020). 33030152 10.1039/d0cp03414b

[13] Nikolic, M. et al. Combinatorial neutron imaging methods for hydrogenation catalysts. *Phys. Chem. Chem. Phys.* **24**, 27394 (2022). 36331375 10.1039/d2cp03863c

[14] Nikolic, M. et al. Operando neutron imaging. *Chimia* **78**, 333–338 (2024). 38822777 10.2533/chimia.2024.333

[15] Cavaye, H. et al. In situ real-time neutron imaging of gaseous H_2_ adsorption and D_2_ exchange on carbon-supported Pd catalysts. *Chem. Commun.* **59**, 12767–12770 (2023). 10.1039/d3cc03930g 37812072

[16] Shaikhutdinov, Sh. et al. Structure–reactivity relationships on supported metal model catalysts: Adsorption and reaction of ethene and hydrogen on Pd/Al_2_O_3_/NiAl (110). *J. Catal.* **200**, 330–339 (2001).

[17] Bugaev, A. L. et al. Hydrogenation of ethylene over palladium: Evolution of the catalyst structure by operando synchrotron-based techniques. *Faraday Discussions.* **229**, 197–207 (2021). 33656030 10.1039/c9fd00139e

[18] Vogt, C. & Weckhuysen, B. M. The concept of active site in heterogeneous catalysis. *Nat. Rev. Chem.* **6**, 89–111 (2022). 37117296 10.1038/s41570-021-00340-y

[19] Mao, S. J., Wang, Z., Luo, Q., Lu, B. & Wang, Y. Fast and selective dehydrogenative C-H/C–H arylation using mechanochemistry. *ACS Catal.* **13**, 974–1019 (2023).

[20] Minniti, T., Watanabe, K., Burca, G., Pooley, D. E. & Kockelmann, W. Characterization of the new neutron imaging and materials science facility IMAT. *Nucl. Instrum. Methods Phys. Res. Sect. A* **888**, 184–195 (2018).

[21] Tygier, S. et al. Tomographic reconstruction with Mantid Imaging. *J. Phys. Conf. Ser.* **2605**, 012017 (2023).

[22] Akello-Egwel, D. et al. Mantid imaging. Zenodo. 10.5281/zenodo.4728059 (2020).

[23] Schindelin, J. et al. Fiji: An open-source platform for biological-image analysis. *Nat. Methods* **9**, 676–682. 10.1038/nmeth.2019 (2012). 22743772 10.1038/nmeth.2019 PMC3855844

[24] Campbell, J. W. et al. The application of alumina supported Pd catalysts for high selectivity aniline synthesis catalysis at elevated temperatures: Site-selective chemistry. *Appl. Catal. A General* **670**, 119541 (2024).

[25] Webb, G. The formation and role of carbonaceous residues in metal-catalysed reactions of hydrocarbons. *Catal. Today* **7**, 139–155 (1990).

[26] Lennon, D. et al. The application of neutron imaging to investigate the hydrogenation of nitrobenzene over an alumina-supported palladium catalyst. *STFC ISIS Neutron Muon Source.*10.5286/ISIS.E.RB2310426 (2023).

