# Supplementary material for: The application of neutron imaging to examine ethene hydrogenation over a carbon-supported palladium catalyst
Source: Sci Rep. 2025 Mar 12;15:8579. doi: 10.1038/s41598-025-91179-6 (PMC11904201; doi:10.1038/s41598-025-91179-6)
Supplement: Supplementary file 1 — Supplementary Information 1. [file 41598_2025_91179_MOESM1_ESM.docx]

**Supporting Information**

Video S1: Video presentation showing a continuous series of selected neutron radiographs of the catalyst cell over the period when the ethene was switched in to the He/H_2_ gas feed (Figure 3(b)).

The data used to plot the grey value figures (Figures 1(b)(c), 2(b)(c), 3(b)(c), 5(b)(c), 6(b)(c), 7(b)(c)) are available as electronic supplementary material in both raw and smoothed versions.
